# An Update on the Potential Application of Herbal Medicine in Promoting Angiogenesis

**DOI:** 10.3389/fphar.2022.928817

**Published:** 2022-07-19

**Authors:** Jingjing Li, Renkai Li, Xiaoping Wu, Chengwen Zheng, Polly Ho-Ting Shiu, Panthakarn Rangsinth, Simon Ming-Yuen Lee, George Pak-Heng Leung

**Affiliations:** ^1^ Department of Rehabilitation Sciences, Faculty of Health and Social Sciences, Hong Kong Polytechnic University, Kowloon, Hong Kong SAR, China; ^2^ Department of Pharmacology and Pharmacy, The University of Hong Kong, Pokfulam, Hong Kong SAR, China; ^3^ State Key Laboratory of Quality Research in Chinese Medicine and Institute of Chinese Medical Sciences, University of Macau, Taipa Macao SAR, China

**Keywords:** herbal medicine, phytochemicals, pro-angiogenic, wound healing, ischemic diseases

## Abstract

Angiogenesis, the formation of new capillaries from pre-existing vascular networks, plays an important role in many physiological and pathological processes. The use of pro-angiogenic agents has been proposed as an attractive approach for promoting wound healing and treating vascular insufficiency-related problems, such as ischemic heart disease and stroke, which are the leading causes of death worldwide. Traditional herbal medicine has a long history; however, there is still a need for more in-depth studies and evidence-based confirmation from controlled and validated trials. Many *in vitro* and *in vivo* studies have reported that herbal medicines and their bioactive ingredients exert pro-angiogenic activity. The most frequently studied pro-angiogenic phytochemicals include ginsenosides from *Panax notoginseng*, astragalosides and calycosin from Radix Astragali, salvianolic acid B from *Salvia miltiorrhiza*, paeoniflorin from Radix Paeoniae, ilexsaponin A1 from *Ilex pubescens*, ferulic acid from *Angelica sinensis*, and puerarin from Radix puerariae. This review summarizes the progress in research on these phytochemicals, particularly those related to pro-angiogenic mechanisms and applications in ischemic diseases, tissue repair, and wound healing. In addition, an outline of their limitations and challenges during drug development is presented.

## Introduction

Angiogenesis is the formation of new blood vessels. It plays a critical role in regulating a wide range of physiological activities such as embryonic development, menstrual cycle, wound healing, tissue repair after surgery, and traumatic injury (Carmeliet, 2003). Angiogenesis is tightly regulated by several stimulators and inhibitors. An imbalance between these stimulators and inhibitors is implicated in diseases including cancer, cardio-cerebral diseases, age-related macular degeneration, arthritis, psoriasis, obesity, and even asthma. Therefore, the manipulation of angiogenesis is considered an attractive and promising approach for curing these pathological conditions.

### Process of Angiogenesis

Angiogenesis is categorized into two major types: sprouting and intussusceptive angiogenesis (Kurz, 2000). In sprouting angiogenesis, the processes of endothelial cell migration and proliferation, sprout fusion, and lumen formation are precisely coordinated (Franco et al., 2009). When endothelial cells are stimulated by pro-angiogenic factors, they produce proteases that break down the surrounding basement membrane, allowing them to move out of the pre-existing blood vessel lumen. The endothelial cells proliferate extensively in the surrounding matrix. Thus, solid sprouts are formed. After recruiting mural cells (i.e., pericytes and smooth muscle cells), the sprouts are ultimately transformed into mature blood vessels (Carmeliet, 2000; Duran et al., 2017).

In intussusceptive angiogenesis, blood vessels are divided into two new types (Burri et al., 2004). This process starts with the projection of opposing capillary walls inside the lumen of the original blood vessel, and inter-endothelial contact is built up. Afterward, growth factors and cells such as fibroblasts and pericytes enter. A pillar is formed between the two new vessels. This pillar eventually disappears and the two independent blood vessels split (Burri et al., 2004).

Sprouting and intussusceptive angiogenesis are functionally distinct. Sprouting angiogenesis promotes the formation of blood vessels in previously unvascularized tissues, whereas intussusceptive angiogenesis allows for the rapid-recovery adaption of existing microvascular networks (Heil et al., 2006). Sprouting and intussusceptive angiogenesis can occur concurrently during the development of certain organs or tissues, such as the lung, heart, and yolk sac (Kurz, 2000). It has been suggested that sprouting angiogenesis is more common during brain development, whereas intussusceptive angiogenesis is predominant in the lungs (Kurz, 2000; Burri and Djonov, 2002). Interestingly, increasing evidence has shown that intussusceptive angiogenesis is more important than sprouting angiogenesis in embryonic development. This is because there are inadequate resources to produce large numbers of endothelial cells during embryonic development. Intussusceptive angiogenesis can rapidly and massively increase the number of blood vessels without a corresponding increase in endothelial cells (Carmeliet, 2003; Djonov et al., 2003).

### Regulation of Angiogenesis

Endogenous pro-angiogenic and anti-angiogenic factors are listed in Table 1. VEGF is the most representative pro-angiogenic factor, inducing endothelial cell survival, proliferation, migration, invasion, lumen formation, and blood vessel maturation (Cébe-Suarez et al., 2006). Hypoxia-inducible factor (HIF)-1 is a major driver of angiogenesis. HIF-1 comprises an oxygen-regulated α-subunit and ubiquitously expressed *ß*-subunit. Under hypoxia, the hydroxylation of HIF-1*α* is inhibited. HIF-1α, therefore, accumulates and translocates to the nucleus, where it forms a dimer with HIF-1*β* and binds to the VEGF promoter. Thus, the expression of VEGF mRNA and the subsequent release of VEGF are increased.

**TABLE 1:** List of endogenous angiogenic stimulators and inhibitors.

Endogenous angiogenic stimulators	Endogenous angiogenic inhibitors
Adrenomedullin Angiogenin Angiopoietin-1 Fibroblast growth factor-1 Fibroblast growth factor-2 Follistatin Granulocyte-colony-stimulating factor Hepatocyte growth factor/scatter factor Interleukin-3 Interleukin-8 Intermedin Keratinocyte growth factor Leptin Midkine Neuregulin Osteogenic protein-1 Placental growth factor Platelet-derived endothelial-cell growth factor Platelet-derived growth factor Pleiotrophin Progranulin Proliferin Transforming growth factor-α Transforming growth factor-β Tumor necrosis factor-α Vascular endothelial growth factor Vascular permeability factor	2-Methoxyestradiol Angiostatin Antithrombin III fragment Arresten Canstatin Chondromodulin I Connective tissue growth factor Decorin Endorepellin Endostatin Interferon-inducible protein-10 Interferons-*α*, *β*, and *γ* Interleukin-10 Interleukin-12 Interleukin-4 Kringle 5 Maspin Osteopontin cleavage product Pigment epithelium-derived factor Plasminogen activator inhibitor Platelet factor-4 Prothrombin kringle 2 Restin Soluble fms-like tyrosine kinase-1 Tetrahydrocortisol-S Thrombospondin-1 and -2 Tissue inhibitors of matrix metalloproteinases Troponin-1 Tumstatin Vascular endothelial growth inhibitor Vasostatin

Mechanistically, VEGF regulates the secretion and activation of enzymes and factors [e.g. matrix metallopeptidases (MMP) and plasminogen activators] involved in the degradation of the extracellular matrix, a critical step in angiogenesis (Carmeliet, 2000). Moreover, VEGF protects newly formed blood vessels against apoptosis by promoting the anti-apoptotic factors B-cell lymphoma (Bcl)-2 and survivin (Karaman et al., 2018). Basic information on VEGF family members, such as splice variants, sources, receptors, and functions, is given in Table 2.

**TABLE 2:** Members of the VEGF family and their biological functions.

Categories	Splice variants	Sources	Receptors	Biological functions
VEGF-A	VEGF_121_, VEGF_145_, VEGF_165_, VEGF_183_, VEGF_189_, VEGF_206_	Human, mouse, rat, bovine, dog, zebrafish, etc.	VEGFR-1, VEGFR-2, NRP-1, NRP-2	Angiogenesis, vasculogenesis
VEGF-B	VEGF-B_167_, VEGF-B_186_	Human, mouse, bovine, rat, etc.	VEGFR-1, NRP-1	Embryonic angiogenesis
VEGF-C	None	Human, mouse, bovine, zebrafish, rat, etc.	VEGFR-2, VEGFR-3	Angiogenesis, lymphangiogenesis
VEGF-D	None	Human, mouse, bovine, rat, etc.	VEGFR-2, VEGFR-3	Angiogenesis, lymphangiogenesis
VEGF-E	None	Orf virus	VEGFR-2, NRP-1	Angiogenesis
VEGF-F	None	Snake venom	VEGFR-2	Angiogenesis
PIGF	PIGF-1, PIGF-2, PIGF-3, PIGF-4	Human, mouse, bovine, rat, etc.	VEGFR-1, NRP-1, NRP-2	Angiogenesis, vasculogenesis

**FIGURE 1:**
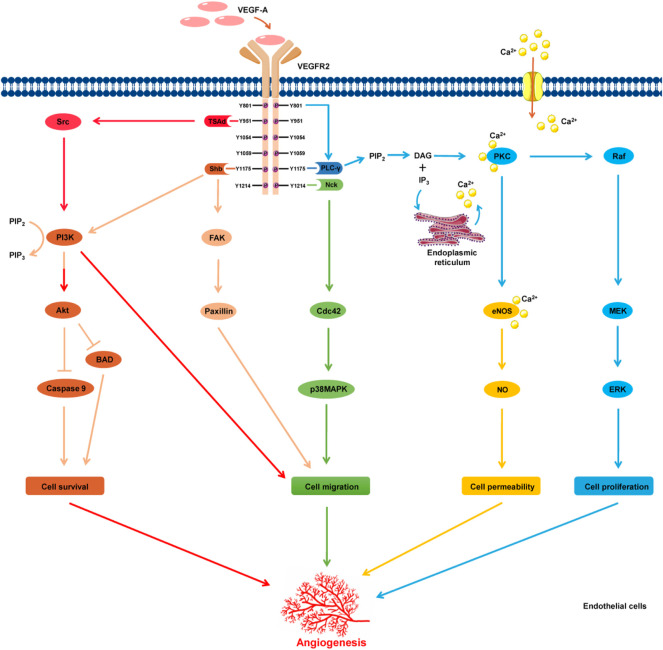
Schematic illustration of VEGFR-2 signaling pathways. Stimulation of VEGFR-2 by VEGF-A induces dimerization and autophosphorylation of specific intracellular tyrosine residues. Subsequently, the activation of the downstream signal transduction pathways induces the proliferation, migration, and survival of endothelial cells, and increases vascular permeability.

VEGF receptors (VEGFR) comprise extracellular domains with seven immunoglobulin-like subdomains, a single transmembrane spanning domain, and an intracellular tyrosine kinase domain (Hoeben et al., 2004). The VEGFR family comprises three members. Among these, VEGFR-2 is the most important receptor involved in VEGF-induced angiogenesis (Holmes et al., 2007; Wang et al., 2020). On stimulation with VEGFR-2, the subsequent dimerization of two receptor monomers causes phosphorylation of tyrosine residues in the intracellular domain, which induces the activation of a series of signaling pathways, leading to the formation of new blood vessels (Peach et al., 2018; Fallah et al., 2019; Wang et al., 2020) (Figure 1). The downstream signaling pathways of VEGFR-2 include the protein kinase C (PKC)/proto-oncogene serine/threonine-protein kinase (Raf)/mitogen-activated protein kinase (MEK)/extracellular signal-regulated kinase (ERK) signaling pathway, which is related to endothelial cell proliferation; the SH2 domain containing adapter protein B (Shb)/focal adhesion kinase (FAK) and cell division control protein 42 homolog (Cdc42)/p38 MAPK signaling pathways, which are responsible for endothelial cell migration; inhibition of the Bcl-2 associated death (BAD) promoter and caspase-9 expression by the PI3K/protein kinase B (Akt)-dependent pathway, which is associated with endothelial cell survival; and PKC/endothelial nitric oxide synthase (eNOS), which catalyzes the production of nitric oxide, increasing vascular permeability.

### Relationship Between Angiogenesis and Diseases

Increased rates of angiogenesis are associated with the pathogenesis of diseases such as cancer (Weis and Cheresh, 2011), psoriasis (Weidemann et al., 2013), rheumatoid arthritis (Koch and Distler, 2007), diabetic retinopathy (Behl and Kotwani, 2015), and age-related macular degeneration (Ng and Adamis, 2005). VEGF is implicated in the pathology of these conditions. Anti-angiogenic therapy has been used for the treatment of cancer and macular degeneration (Carmeliet, 2005; Ng and Adamis, 2005; Liu et al., 2006; Copur, 2008; Wilhelm et al., 2008; Escudier et al., 2009; Capdevila et al., 2012; Al-Ostoot et al., 2021). In addition to VEGF, deficiency of leucine-rich α-2-glycoprotein 1 (LRG1), a novel pro-angiogenic factor, also leads to several pathophysiological processes such as retinopathy and diabetic wounds. The mechanism involves the TGF-*β* accessory receptor, endoglin, apoptosis-activating factor-1, and HIF-1*α* stability (Wang et al., 2013a; Gao et al., 2020; Jemmerson et al., 2021). Angiopoietins and vasohibin-2 are two additional angiogenic factors involved in pathogenesis. It has been reported that the imbalance of the angiopoietin-1 and angiopoietin-2 ratio and deficiency of vasohibin-2 play a role in the pathogenesis of glomerular diseases (Zhang et al., 2020).

In contrast, insufficient angiogenesis leads to diseases such as ischemic heart failure and stroke, neurodegeneration, osteoporosis, respiratory distress, preeclampsia, endometriosis, and ovarian hyperstimulation syndrome (Carmeliet, 2003). Theoretically, pro-angiogenic agents can improve blood perfusion, transport survival factors to ischemic tissues, mobilize regenerative stem cell populations, and ultimately restore tissue functions (Majewska and Gendaszewska-Darmach, 2011; Mitsos et al., 2012). Pro-angiogenic therapy has been suggested as a promising approach for wound healing and tissue repair. A clinical trial has been conducted on patients with coronary artery disease, and their conditions can be improved after the injection of human VEGF (Sarkar et al., 2001). In addition, improved vascularization of regenerated tissue is necessary after tissue implantation and subsequent major operations. The incorporation of VEGF and FGF may increase the cure and surgical success rates in critically ill patients (Nomi et al., 2002).

## Herbal Medicine and Phytochemicals as Novel Pro-Angiogenic Agents and Their Potential Implications

**FIGURE 2:**
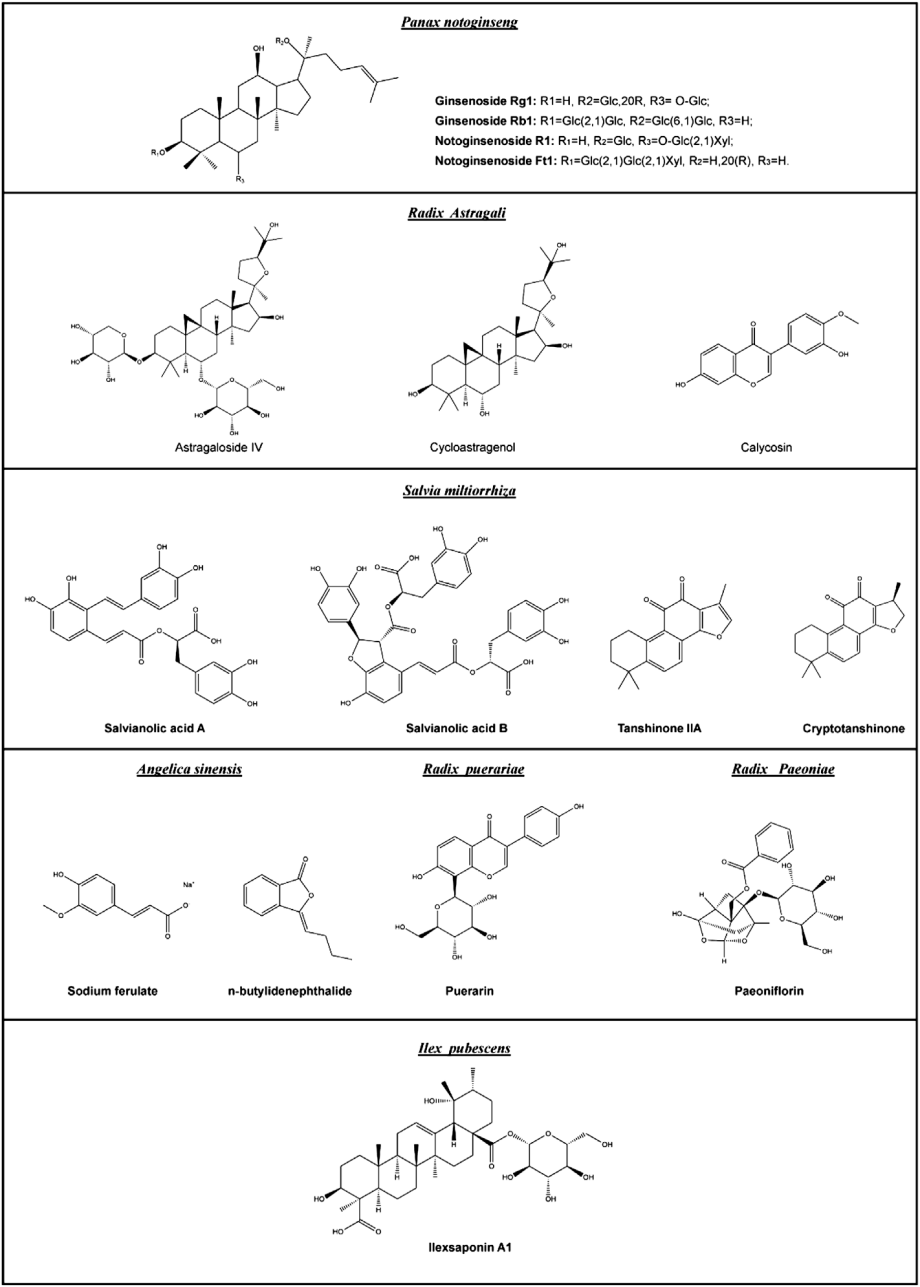
Chemical structures of the major pro-angiogenic phytochemicals mentioned in this review.

Native pro-angiogenic agents inside the body, such as VEGF and FGF, are not ideal options for pro-angiogenic therapy because their therapeutic efficacy is often transient. This is related to the development of drug resistance and degradation over time. Small-molecule drugs may be a better alternative, for many reasons. First, they are more stable than proteins under a wider range of conditions. Second, they are less expensive in terms of production costs. Third, they may act on multiple targets, reducing the risk of drug resistance. Many small-molecule drugs used clinically are derived either directly or indirectly from plants. According to the National Administration of Traditional Chinese Medicine, the advantages of traditional Chinese medicine are particularly prominent in treating 95 types of diseases, including diseases closely related to angiogenesis, such as ischemic diseases and diabetic ulcers (National Administration of Traditional Chinese Medicine, 2019). Therefore, many studies have attempted to explore the potential pro-angiogenic effects of herbal medicines and their active ingredients. The following examples of herbal medicines were chosen because they are better studied and their pro-angiogenic effects are more widely reported. They have been used for a long time; therefore, their efficacy and safety are well recognized. The chemical structures, pharmacology, and potential applications of phytochemicals in these herbal medicines are given in Figure 2 and Table 3.

**TABLE 3:** Summary of pro-angiogenic phytochemicals and their potential applications.

Species	Ingredients	Pro-angiogenic mechanisms	Experimental models	Potential applications	Ref.
*Panax notoginseng*	PNS	N/A	LAD ligation mice	Myocardial infarction	Zhang et al. (2016)
		Upregulation of AMPK- and eNOS-dependent pathways	HUVECs	N/A	Wang et al. (2014)
	Notoginsenoside R1	Activation of VEGF/VEGFR2 and PI3K-Akt-eNOS pathways	HUVECs, zebrafish	N/A	Zhang et al. (2009)
	Notoginsenoside Ft1	Stimulation of HIF-1α-mediated VEGF secretion and upregulation of PI3K/AKT and Raf/MEK/ERK pathways	HUVECs, Matrigel plugs, ear wound-healing mice	Wound healing	Zhang et al. (2012)
	Ginsenoside Rg1	Activation of NO signaling via suppressing miR-23a/IRF-1 axis	HUVECs, streptozotocin-induced diabetic rats	Diabetic wound healing	Zhang et al. (2011)
Radix Astragali	Radix Astragali extract	Activation of VEGF and PI3K/Akt/eNOS pathways	HUVECs	N/A	Li et al. (2021a)
	Astragaloside IV	Upregulation of VEGF expression	HUVECs, zebrafish	N/A	Chen et al. (2012)
		Stimulation of the HIF-1α-mediated PI3K/Akt pathway	HUVECs	N/A	Huang et al. (2021)
		Activation of HIF-1α SUMOylation	HUVECs	N/A	Lee et al. (2018)
		Activation of the SUMOylation pathway	HUVECs, diabetic wound-healing rats	Diabetic wound healing	Li et al. (2012)
		Upregulation of the PKD1/HDAC5/VEGF pathway	LAD ligation rats	Myocardial infarction	Luo et al. (2016)
		Activation of the miRNA-210-mediated HIF/VEGF/Notch pathway	HUVECs, MCAO rats	Ischemic stroke	Wang et al. (2021d)
		N/A	EPCs, wound healing mice	Wound healing	Tang et al. (2010)
		Activation of AKT/GSK-3β/β-catenin signaling	Ea.hy926 cells, BMSCs, DO rats	Orthopedic surgery, and oral and maxillofacial surgery	Irmak et al. (2018)
	Calycosin	Stimulation of VEGF, FGF, and EGF signals	HUVECs, zebrafish	N/A	Li et al. (2014)
		Activation of ER and the MAPK signaling pathway	HUVECs, zebrafish	N/A	Li et al. (2020a)
*Salvia miltiorrhiza*	*Salvia miltiorrhiza* extract	Upregulation of VEGF and VEGF receptor gene expressions	Murine endothelial cells	N/A	Liang et al. (2009)
		Elevation of HIF-1α and VEGF expressions	LAD ligation mice	Myocardial infarction	Chen et al. (2014)
		Upregulation of VEGF, BDNF, and eNOS expressions	MCAO mice	Ischemic stroke	He and Shen, (2014)
		N/A	Burn wound-healing rats	Burn wounds	Xu et al. (2009)
	Salvianolic acids	N/A	EPCs, CAM model	N/A	Zhang et al. (2014)
	Salvianolic acid A	Upregulation of VEGF, VEGFR-2, and MMP-9 protein expressions	EPCs, LAD ligation rats	Myocardial infarction	Song et al. (2020)
	Salvianolic acid B	Activation of the VEGF/VEGFR2 signaling pathway	HUVECs, streptozotocin-induced diabetic mice	Diabetic cardiomyopathy	Li et al. (2016)
	Tanshinone IIA	Upregulation of HIF-1α and VEGF protein expressions	LAD ligation rats	Myocardial infarction	Zhao et al. (2006)
	An imidazole-tanshinone	Activation of VEGF/FGF-Src and PI3K-P38 MAPK signaling pathways	Zebrafish	N/A	Hsiao et al. (2012)
	Cryptotanshinone	Upregulation of VEGF, Ang-1, and eNOS expressions	Diabetic wound-healing mice	Diabetic wounds	Zhang et al. (2006)
*Angelica sinensis*	*Angelica sinensis* extract	Activation of VEGF, p38 and JNK 1/2 protein expressions	HUVECs, zebrafish	N/A	Zhang et al. (2008)
		Stimulation of p38/HIF-1α/VEGF-A signaling pathway	MCAO rats	Ischemic stroke	Tang et al. (2014)
	Sodium ferulate, n- ferulate butylidenephthalide	Enhancement of astrocyte-derived VEGF and BDNF expression via the activation of the AKT/mTOR pathway	BM-MSCs, MCAO rats	Ischemic stroke	Xu et al. (2019)
Radix puerariae	Puerarin	Upregulation of VEGF, HIF-1α, Akt, and eNOS protein expressions	LAD ligation rats	Myocardial infarction	Dong et al. (2020)
		Activation of the p42/44 MAPK signaling pathway	MECs, spontaneously hypertensive rats	Ischemic stroke	Fang et al. (2020)
Radix Paeoniae	Paeoniflorin	Upregulation of VEGFR1 and VEGFR2 gene expressions	HUVECs, zebrafish	N/A	Li et al. (2017)
		Activation of the VEGF/VEGFR-2 signaling pathway	EPCs, MCAO rats	Ischemic stroke	Yan et al. (2017)
		Upregulation of VEGF protein expression	Streptozotocin-induced diabetic rats	Diabetic wounds	Shang et al. (2012)
*Ilex pubescens*	Ilexsaponin A1	Activation of Akt/mTOR, MAPK/ERK, and FAK signaling pathways	HUVECs, zebrafish	N/A	Kant et al. (2021)

### Panax notoginseng

The root of *Panax notoginseng* (Burkill) F.H. Chen, also known as San Qi in Chinese, is a traditional herbal medicine used in Asia to treat blood stasis and promote blood circulation (Yu et al., 2007; Cheung et al., 2011). Moreover, *P. notoginseng* has been proposed for the treatment of cardiovascular diseases, inflammatory diseases, and traumatic injuries. The basic active ingredients in *P. notoginseng* are saponins (Liu et al., 2014); more than 60 saponins have been identified in this plant (Wang et al., 2013b), of which ginsenoside Rg1, ginsenoside Rb1, and notoginsenoside R1 are the major *P. notoginseng* saponins (PNS). These compounds possess promising angiogenic effects *in vitro* and *in vivo* at multiple sites of action (Hong et al., 2009; Zheng et al., 2013a). Interestingly, the saponins extracted from the flowers of *P. notoginseng* exhibit similar pro-angiogenic properties, although the types of saponins may be different from those in the roots (Yang et al., 2016a).

It has been reported that PNS can rescue myocardial infarct size and cardiac function in left anterior descending coronary artery ligation-operated mice, an *in vivo* model of acute myocardial infarction. PNS inhibits platelet aggregation and enhances angiogenesis in response to myocardial infarction injury (Wang et al., 2021a). *In vitro* studies have shown that PNS promotes tube formation in endothelial cells through adenosine monophosphate kinase- and eNOS-dependent signaling pathways (Wang et al., 2017). Moreover, notoginsenoside R1 stimulates the proliferation, migration, and tube formation of endothelial cells. It also exerts pro-angiogenic effects in zebrafish models. Its pro-angiogenic action is mediated through the activation of the VEGF-KDR/Flk-1 and PI3K-Akt-eNOS signaling pathways (Yang et al., 2016b). Another saponin, notoginsenoside Ft1, increases the release of VEGF via the HIF-1α-dependent pathway. In addition, notoginsenoside Ft1 induces the activation of PI3K/Akt and Raf/MEK/ERK signaling pathways, which are crucial in the process of angiogenesis (Shen et al., 2012).

The active ingredients of *Panax notoginseng* have promising effects on wound healing. In streptozotocin-induced diabetic rats, ginsenoside Rg1 promoted angiogenesis during wound healing. This is probably mediated through the reduction of miR-23a levels, which removes its inhibitory regulation on interferon regulatory factor-1. Subsequently, interferon regulatory factor-1 activates inducible nitric oxide synthase (iNOS). The increased production of NO increases VEGF expression, proliferation, anti-apoptotic efficacy, and the migration ability of endothelial cells (Cai et al., 2019).

Interestingly, the PNS acts not only on endothelial cells but also on fibroblasts, another important cell type involved in angiogenesis and wound healing. The PNS promotes the proliferation and migration of fibroblasts and increases the expression of collagen and fibronectin. Further mechanistic studies indicate that the PNS plays a role in the phosphorylation of PI3K, Akt, and ERK-dependent pathways (Yu et al., 2015). Ginsenoside Rb1 improved the wound healing rate in a rat model of second-degree burn injury, probably due to the upregulation of PDGF-BB, PDGFR-*β*, and FGF-2 expression (Zhang et al., 2021). In human dermal fibroblasts, notoginsenoside Ft1 increases cell proliferation and collagen production via the PI3K/Akt/mechanistic target of the rapamycin (mTOR) signaling pathway. Notoginsenoside Ft1 shortens the wound closure time in an excisional wound-splinting model established in db/db diabetic mice by promoting fibroblast proliferation and attenuating the inflammatory response (Zhang et al., 2016).

Although the pro-angiogenic effects of PNS and their potential applications have been widely proposed, clinical data are relatively rare. Interestingly, a clinical study reported that a combination of granulocyte colony-stimulating factor (G-CSF)-mobilized peripheral blood mononuclear cell (PBMNC) transplantation and PNS is useful for the treatment of unreconstructable critical limb ischemia (Wang et al., 2014). The combination resulted in a greater reduction in the ulcer area and severity of rest pain than PBMNC transplantation alone. Improvements in parameters such as ankle-brachial index, transcutaneous oxygen pressure, and walking distance were significantly improved after combining PBMNC and PNS.

### Radix Astragali

Radix Astragali, the dried root of *Astragalus mongholicus* Bunge, is also known in Chinese as Huang Qi. It is one of the most popular traditional herbal medicines used to reinforce vital energy. It possesses cardiotonic, hepatoprotective, hypotensive, immunostimulatory, anti-aging, anti-oxidative, antidiabetic, and anti-inflammatory activities. The pro-angiogenic potential of Radix Astragali is reflected in a work showing that Radix Astragali extract can stimulate the proliferation, migration, and tube formation of endothelial cells. The mechanism of action involves the upregulation of VEGF and stimulation of the PI3K-Akt-eNOS pathway (Zhang et al., 2009).

Astragaloside IV (AS-IV) is one of the active ingredients responsible for the pro-angiogenic effects of Radix Astragali. AS-IV can reproduce the effects of Radix Astragali extract on endothelial cells, and it has been shown to rescue blood vessel loss in zebrafish models (Zhang et al., 2012). In terms of the mechanism of action, AS-IV increases HIF-1*α* through the PI3K/Akt pathway in endothelial cells, and the increase in HIF-1*α* stimulates downstream VEGF signaling pathways and regulates angiogenesis in a hypoxic environment (Zhang et al., 2011). In addition, AS-IV stimulates endothelial cells to produce small ubiquitin-related modifier 1, which stabilizes the HIF-1*α*/VEGF pathway and improves angiogenesis (Wang et al., 2021b; Wang et al., 2021c).

The pro-angiogenic effect of AS-IV may be useful in the treatment of myocardial infarction. In rat models of myocardial infarction established by ligation of the left anterior descending artery, AS-IV significantly improved the morphology of the myocardium and the number of new blood vessels. The cardioprotective effect of AS-IV is mediated by its pro-angiogenic effect through the protein kinase D1-histone deacetylase 5-VEGF pathway (Yang et al., 2019). Apart from its cardioprotective effect, AS-IV promotes cerebral protection against ischemic brain injury via its pro-angiogenic action. The specific mechanism involves the activation of the HIF/VEGF/Notch signaling pathway via miRNA-210 (Liang et al., 2020). Interestingly, in addition to the pro-angiogenic effect, AS-IV improves ischemic brain tissue repair by promoting neurogenesis and neurological functional recovery, which is partially mediated by transforming microglia/macrophages from the M1 to M2 phenotype in a PPARγ-dependent manner (Li et al., 2021a).

AS-IV has been reported to be effective in skin wound repair, leading to significant improvements in wound closure, collagen synthesis, and skin tensile strength recovery (Chen et al., 2012; Zhang et al., 2012). When cultured human umbilical cord blood-derived endothelial progenitor cells are treated with AS-IV cells and then combined with human blood plasma gel, the application of this gel can repair full-thickness skin defects in a mouse model (Huang et al., 2021). In addition to its action on endothelial cells, AS-IV and cycloastragenol-6-O-beta-D-glucoside (the major metabolite of astragalosides) increase the proliferation and migration of skin cells via activation of the epidermal growth factor (EGFR)/ERK signaling pathway, resulting in the improvement of wound healing (Lee et al., 2018). Moreover, AS-IV mediates keratinocyte proliferation and migration by downregulating *ß*-catenin (Li et al., 2012). AS-IV also augments skin re-epithelialization and enhances matrix synthesis. Finally, AS-IV induces the development of polarized alternatively activated macrophages, which may reduce inflammation and facilitate wound healing (Luo et al., 2016).

It is interesting to note that AS-IV accelerates bone regeneration by simultaneously enhancing osteogenesis and preosteoclast-induced angiogenesis, partially through Akt/glycogen synthase kinase-3β/β-catenin signaling. Therefore, AS-IV has been proposed as a potential therapeutic agent for patients undergoing distraction osteogenesis treatment (Wang et al., 2021d).

Apart from AS-IV, other saponins in Radix Astragali, such as cycloastragenol, cyclocephaloside I, and cyclocanthoside E increase fibroblast proliferation and migration. These effects were observed to be stronger with cycloastragenol which also possesses a more remarkable wound-healing effect compared with astragaloside IV, with a more regularly organized dermis and more newly formed blood vessels (Sevimli-Gür et al., 2011). Furthermore, calycosin, a major isoflavonoid isolated from Radix Astragali, possesses pro-angiogenic effects. Calycosin induces angiogenesis in cultured endothelial cells and zebrafish models by upregulating VEGF, VEGFR-1, and VEGFR-2 mRNA levels. In addition to the VEGF-dependent pathway, calycosin also modulates the FGF and EGFR signaling pathways (Li et al., 2011). Interestingly, calycosin is similar to other selective estrogen receptor modulators, such as raloxifene and tamoxifen, in the sense that it has selective potency and affinity for estrogen receptors α and *ß*. Through its interaction with estrogen receptors, the subsequent activation of the MAPK-dependent pathway leads to a pro-angiogenic effect (Tang et al., 2010).

### Salvia miltiorrhiza

*Salvia miltiorrhiza* Bunge, also known as Dan Shen in Chinese, is a widely used Chinese herbal medicine, particularly for the treatment of cardiovascular and trauma-related diseases. The crude extract of *S. miltiorrhiza* enhances the growth and differentiation of endothelial cells, probably through the upregulation of MMP-2, VEGF, and VEGFR-2 gene expression (Lay et al., 2003). In a mouse model of left anterior descending coronary artery ligation, *S. miltiorrhiza* treatment partially rescued the angiogenesis defect and heart failure with an increase in the mRNA and protein expression levels of HIF-1α and VEGF-A (Ai et al., 2015). In a rat model, *S. miltiorrhiza* decreased the amount of necrosis in burn wounds by increasing tissue perfusion and neovascularization. This is promising because burn healing is a complicated process and most treatments are ineffective (Irmak et al., 2018). Salvianolic acid tanshinone IIA is the major active ingredient of *S. miltiorrhiza*.

Salvianolic acids increase the number of endothelial progenitor cells and promote endothelial progenitor cell migration, adhesion, and vasculogenesis (Li et al., 2010). Danshensu [(R)-3- (3, 4-Dihydroxyphenyl)-2-hydroxypropanoic acid] has the basic chemical structure of various salvianolic acids. Salvianolic acid A is formed by one danshensu molecule and two caffeic acid molecules. Salvianolic acid B is formed by three danshensu molecules and one caffeic acid molecule. Sodium danshensu significantly increased the newly formed arteries and the diameter of the collateral arteries, leading to enhanced local cerebral blood flow recovery after a stroke. In addition to its effect on angiogenesis, sodium danshensu improves stroke recovery by stimulating neurogenesis in the post-ischemic brain. The mechanism of action involves the increased expression of VEGF, stromal-derived factor-1, brain-derived neurotrophic factor (BDNF), and eNOS in the peri-infarct region (Wei et al., 2018). Salvianolic acid A reduced the infarct size and increased the capillary density in the infarct region in rat myocardial infarction models. This effect is attributed to the increased formation of VEGF, VEGFR-2, and MMP-9, and the promotion of the number and migration capacity of endothelial progenitor cells (Li et al., 2014).

The pro-angiogenic effects and potential applications of salvianolic acid B have been widely studied. Salvianolic acid B enhances the growth and differentiation of endothelial cells (Lay et al., 2003). It also ameliorates left ventricular dysfunction in the hearts of streptozotocin-induced diabetic mice (Li et al., 2020a). Salvianolic acid B promotes angiogenesis in the heart by increasing the expression of VEGFR-2 and VEGF-A. Moreover, it reduces hyperglycemia-induced insulin-like growth factor-binding protein-3 expression, induces ERK and Akt activities, and activates VEGFR-2/VEGFA signaling in endothelial cells (Li et al., 2020a).

Although direct evidence is still unavailable, many studies have indicated that salvianolic acid B is a promising candidate for accelerating wound healing. In addition to its action on angiogenesis, salvianolic acid B inhibits the activities of human MMP-1, MMP-2, and MMP-9. The effects of salvianolic acid B on MMPs are one of the mechanisms accounting for the effects of *Salvia miltiorrhiza* on chronic wounds because MMPs are a family of endopeptidases that can degrade extracellular matrix components. This proteolytic property is important during wound healing to remove debris and facilitate cell migration (Liang et al., 2009). Moreover, salvianolic acid B enhances proliferation and collagen production in human skin cells (Chen et al., 2014), which may accelerate wound healing. Interestingly, salvianolic acid B has been shown to accelerate early-stage bone fracture healing. In a rat tibial fracture model, X-ray imaging and histological data showed that the salvianolic acid B treatment group had better callus growth and improved bone remodeling. Increased alkaline phosphatase activity is also involved in the healing process (He and Shen, 2014).

Tanshinone IIA elicits a significant cardioprotective effect in rats with myocardial infarction by improving heart function, reducing infarct size, and increasing the survival rate. Tanshinone IIA promotes angiogenesis and upregulates VEGF expression in myocardial infarction by enhancing HIF-1α mRNA expression (Xu et al., 2009). In addition to tanshinone IIA, 10 new imidazole-tanshinones and one oxazole-tanshinone have been isolated, and their angiogenesis activities were determined in a zebrafish model. Interestingly, one of the imidazole-tanshinones exhibited potent vascular protective and restorative activity with an EC_50_ value of 0.026 µM, which is very potent. The mechanism underlying its pro-angiogenesis effect involves the VEGF/FGF-Src-MAPK and PI3K-P38 MAPK signaling pathways (Zhang et al., 2014). Cryptotanshinone is a diterpene quinone compound found in *Salvia miltiorrhiza*. In an excisional wound-splinting model in diabetic mice, cryptotanshinone accelerated wound closure. A significant increase in re-epithelialization and granulation tissue formation was observed. Mechanistically, cryptotanshinone suppressed leukocyte infiltration and chemokine C-X-C motif ligand-1 and -2 expressions. In addition, cryptotanshinone increases the blood vessel density, expression levels of VEGF and Ang-1, and activation of eNOS. In addition, extracellular matrix remodeling is enhanced by cryptotanshinone by promoting fibroblast transformation and inhibiting MMP-2 and MMP-9. These findings indicate that the wound-healing effect of cryptotanshinone is mediated by a combined modulatory effect on angiogenesis, inflammation, and extracellular matrix remodeling (Song et al., 2020). Finally, a mouse model showed that while cryptotanshinone accelerated wound healing, the scar margins were narrow and there was less collagen deposition in the regenerated tissue. This indicates that cryptotanshinone can prevent and reduce scarring, which may be an extra advantage (Li et al., 2016).

### Angelica sinensis

*Angelica sinensis* (Oliv.) Diels, also known as Dong Quai in Chinese, has been traditionally used to treat blood-related ailments including menstrual cramps, blood deficiencies, uterine disorders, and cardiovascular and cerebrovascular ischemia. The *in vitro* and *in vivo* pro-angiogenic effects of *A. sinensis* have been demonstrated. *A. sinensis* extract stimulates the proliferation, migration, and tube formation of endothelial cells. The extract has been shown to promote the formation of subintestinal vessels in zebrafish models. The pro-angiogenic effects probably involve the increased expression of VEGF, p38, and JNK 1/2 phosphorylation (Lam et al., 2008). Moreover, the *A. sinensis* extract effectively attenuated cerebral infarcts and improved neurological deficits in animal models. *A. sinensis* extract pretreatment provides neuroprotective effects against astrocyte-mediated cerebral infarction by activating pro-angiogenic and anti-apoptotic signaling pathways. The pro-angiogenic and anti-apoptotic effects of the *A. sinensis* extract can be attributed to the activation of the p38 MAPK/HIF-1/VEGF-A signaling pathway and p38 MAPK/HIF-1/VEGF-A/p-BAD-related regulation of the cytochrome c/caspase-3 signaling pathway, respectively, in the cortical ischemic penumbra after reperfusion (Cheng et al., 2017). The active ingredient responsible for the pro-angiogenic effect of *A. sinensis* has not yet been confirmed. Sodium ferulate and n-butylidenephthalide are the two major components of *A. sinensis*. Bone marrow-derived mesenchymal stem/stromal cells (BM-MSCs) have been demonstrated to enhance angiogenesis and the proliferation of reactive astrocytes, which subsequently leads to the amelioration of neurological injury. Interestingly, a combination of sodium ferulate and n-butylidenephthalide with BM-MSCs further enhances the expression of astrocyte-derived VEGF and BDNF, which contribute to angiogenesis after cerebral ischemia, and the underlying mechanism is associated with the activation of the astrocytic Akt/mTOR signaling pathway (Zhang et al., 2017).

A low molecular weight fraction of *Angelica sinensis* possesses strong wound-healing activity in a diabetic mouse wound-healing model and a human/severe combined immunodeficiency mouse chimera wound-healing model. In both models, this fraction of *A. sinensis* was compared favorably with becaplermin, which is a human PDGF approved by the Food and Drug Administration (FDA) for wound-healing treatment. Mechanistically, this fraction strongly stimulates endothelial cell proliferation and tri-dimensional endothelial cell network formation, which are important in the process of angiogenesis. This fraction stimulates the proliferation of human dermal fibroblasts and type I collagen secretion (Zhao et al., 2006). The mechanism of the wound-healing effects of the *A. sinensis* extract and its active water-soluble component, ferulic acid, has been studied using proteomic and biochemical analyses. The treatment of fibroblasts with *A. sinensis* extract modulated the upregulation of proteins associated with cell growth, metabolism, calcium ion regulation, and anti-apoptosis. In contrast, ferulic acid has the unique ability to modulate the upregulation of proteins associated with mobility, anti-oxidative functions, and calcium ion regulation in fibroblasts (Hsiao et al., 2012).

### Radix Puerariae

Radix puerariae lobatae is the dried root of *Pueraria lobata (Wild.) Ohwi*, also known as Ge Gen in China. It is a traditional herbal medicine used for the treatment of a wide range of diseases, including the common cold, influenza, shoulder stiffness, diarrhea, deafness, and cardiovascular disease. The major active ingredient in Radix puerariae is puerarin, which is useful in treating patients with coronary artery disease. Puerarin induces angiogenesis in the non-ischemic and ischemic myocardium, which is one of the mechanisms for reducing the infarct area in animal models. The pro-angiogenic effect of puerarin is due to the induction of gene expression and activation of VEGF, HIF-1α, eNOS synthase, and Akt (Zhang et al., 2006). Interestingly, the cardioprotective effect of puerarin is mediated by the alleviation of oxidative stress and the activation of the PI3K/Akt pathway, which limits endothelial cell migration (Zhang et al., 2008; Li et al., 2020b). Moreover, PKCε and miR-21 contribute to the cardioprotective effects of puerarin by inhibiting apoptosis and oxidative stress in cardiomyocytes (Tang et al., 2014; Xu et al., 2019). Apart from cardioprotection, puerarin prevents stroke by improving microcirculation, which results from an increase in cerebral blood perfusion both by arteriole relaxation and p42/44 MAPK-mediated angiogenesis (Wu et al., 2014).

### Radix Paeoniae

Radix Paeoniae Alba is the dried root of *Paeonia lactiflora* Pall*.*, also named Bai Shao in Chinese, and is used as an herbal medicine for the treatment of various ailments such as liver disease, inflammation, and emotion-related diseases. It is also used to activate blood circulation and remove blood stasis. Paeoniflorin is an active ingredient in Radix Paeoniae. The pro-angiogenic effects of paeoniflorin have been demonstrated in endothelial cells and a zebrafish vascular insufficiency model. Paeoniflorin enhances the proliferation, migration, and tube formation of endothelial cells. Paeoniflorin rescues VEGF tyrosine kinase inhibitor II-induced blood vessel loss in a zebrafish model (Xin et al., 2018). Interestingly, molecular docking studies have revealed that paeoniflorin has an excellent binding ability to PI3K and Akt. A Western blot analysis showed that paeoniflorin suppressed the phosphorylation of PI3K and Akt. This provides clues to the mechanism of the pro-angiogenic action of paeoniflorin (Dong et al., 2020).

The therapeutic application of paeoniflorin in ischemic diseases, particularly those related to the brain, has been proposed. In a rat model of ischemic stroke, paeoniflorin reduced cerebral infarction, alleviated pathological injury, induced the secretion of pro-angiogenic factors, and increased cerebral vascular density. The angiogenic action of paeoniflorin is mediated through the upregulation of VEGF/VEGFR-2 expression (Liu et al., 2021). Moreover, paeoniflorin significantly attenuates cerebral infarction and the severity of intimal hyperplasia in animal models of ischemia/reperfusion injury. Paeoniflorin acts not only on endothelial cells but also on vascular smooth muscle cells. It reduces PDGF-stimulated vascular smooth muscle cell proliferation and migration, probably by modulating the Ras/MAPK/ERK signaling pathway (Chen et al., 2013).

Paeoniflorin improves wound healing in rat models of diabetic foot ulcers. *In vitro* experiments confirmed that paeoniflorin alleviates oxidative stress, increases proliferation and migration, decreases apoptosis, and upregulates VEGF and TGF-β1 expression in endothelial cells (Sun et al., 2020). In addition to its effect on angiogenesis, paeoniflorin accelerates wound healing through collagen deposition (Dong et al., 2020). Furthermore, paeoniflorin exerts anti-inflammatory effects by downregulating interleukin-1β, interleukin-18, and tumor necrosis factor-alpha. Paeoniflorin also decreases expression levels of the chemokine receptor and nuclear factor-kappa B and increases the expression levels of the inhibitor of nuclear factor kappa B. These findings suggest that paeoniflorin significantly attenuates wound inflammation, which may lead to improved wound healing (Sun et al., 2021).

### Ilex pubescens

The roots of *Ilex pubescens* Hook. & Arn., also known as Mao Dong Qing in Chinese, has been used as a traditional herbal medicine for the treatment of stroke, coronary arterial disease, and peripheral vascular diseases. It has been reported that the ethanolic extract of *I. pubescens* promotes cerebral ischemic tolerance and exerts neuroprotective effects. In rats subjected to middle cerebral artery occlusion/reperfusion injury, the ethanolic extract of *I. pubescens* significantly reduces the cerebral infarct volume and increases neurological deficit scores. The underlying mechanism involves the toll-like receptor 4 signaling pathway through the inhibition of the production of proteins or cytokines downstream of the myeloid differentiation factor 88-dependent pathway and the activation of the toll/interleukin-1 receptor domain-containing adapter inducing interferon-β-dependent anti-inflammatory pathways (Fang et al., 2020). Ilexsaponin A1 is a major bioactive ingredient in *Ilex pubescens*. The pro-angiogenic effect of ilexsaponin A1 is reflected in its ability to promote proliferation, migration, invasion, and tube formation of endothelial cells and rescue blood vessel loss in zebrafish models. The mechanism of action probably involves the activation of the Akt/mTOR-, MAPK/ERK-, and FAK-dependent signaling pathways (Li et al., 2017).

The flavonoids in *Ilex pubescens* possess pro-angiogenic effects, which may account for its cerebrovascular protective effect. In a rat model of cerebral ischemia with blood stasis, total flavonoids of *I. pubescens* improved blood circulation, energy metabolism, endogenous anti-oxidative capability, and anti-apoptotic effects, thereby relieving nerve cell injury (Miao et al., 2017). Flavonoids in *I. pubescens* not only reduce damage to brain nerve cells but also significantly reduce the content of nitric oxide in brain homogenates and increase ATP synthetase enzyme activity. Thus, cerebral ischemia-reperfusion injury is improved (Yan et al., 2017). In addition, the neuroprotective effect of flavonoids from *I. pubescens* is linked to the decreased production of pro-inflammatory cytokines such as interleukin-1*β* and tumor necrosis factor-α and increased production of anti-inflammatory cytokines such as interleukin-10 and neurotrophic factors (Fang et al., 2017).

### Limitation of Natural Products and Future Directions

Although the pharmacological actions of phytochemicals have been widely reported, in-depth studies on their pharmacokinetic and pharmacodynamic properties should be conducted. Drug delivery is a critical factor for the success of drug development. In the treatment of ischemic heart or brain diseases, either injections or oral dosage forms are usually used. For the oral route of administration, drug dissolution and absorption rates are affected mainly by the chemical properties of the drugs. Some phytochemicals, such as astragaloside IV, calycosin, tanshinone IIA, and puerarin, are poorly water-soluble, which may lead to slow dissolution and low bioavailability. The use of different salt forms or derivatives of compounds is an easy approach to enhancing drug absorption. For instance, it has been reported that sodium tanshinone IIA sulfonate, a water-soluble derivative of tanshinone IIA, shows a better bioavailability than tanshinone II does (Shang et al., 2012). Similar to tanshinone II, sodium tanshinone IIA sulfonate has a good pro-angiogenic effect, as it can improve perfusion recovery and increase capillary densities in the ischemic hind limbs of diabetic mice (Chen et al., 2019). In addition to drug absorption, the intraluminal metabolic activity occurring in the intestine due to bacterial flora and the first-pass effect due to hepatic metabolism may affect the amount of drugs reaching the systemic circulation. Therefore, it would be useful to develop a derivative that is more resistant to metabolism. In addition, it is critical to study whether these metabolites are bioactive.

For the topical treatment of wound healing, the drug should adhere to the skin for an adequate amount of time, and the drug release rate should be well controlled. It has been reported that ginsenoside Rg1 incorporated into collagen/chitosan–gelatin microsphere scaffolds is advantageous for wound healing and tissue regeneration (Zheng et al., 2013b). Insufficient skin-surface penetration by drugs is another challenge. These problems can be overcome by particle size manipulation through the application of nanotechnology and by encapsulating drugs into nanoformulations. Another advantage of encapsulation is that it allows sustained controlled-release delivery, which is useful for the lengthy wound-healing process (Kant et al., 2021).

Regarding the pharmacodynamics of pro-angiogenic compounds, a more comprehensive understanding of their mechanisms of action is required. For instance, the wound-healing process is related not only to blood vessel formation but also to the proliferation of skin cells and anti-inflammation. Therefore, phytochemicals with bioactivity toward multiple targets are valuable. Furthermore, the structure-activity and dose-effect relationships of phytochemicals should be further explored. Many pro-angiogenic phytochemicals are glycosides. It is important to know whether the number and type of glycosides affect the pro-angiogenic activity. Glycosides may be removed by glycosidases released by the gut flora, so it is necessary to know if aglycones possess pro-angiogenic activities. In addition, the structural modification of phytochemicals may change the binding affinity to their corresponding receptors, thereby affecting the efficiency of the drugs. Finally, the dose-response relationship should be studied because it is plausible that phytochemicals may have different pharmacological effects at different concentrations. Salvianolic acid B is one example; its effects on osteoblast bone formation, angiogenesis, and adipogenesis have been studied in a mouse model of glucocorticoid-induced osteoporosis (Cui et al., 2012). A low dose of salvianolic acid B prevents glucocorticoid-induced cancellous bone loss and increases adipogenesis by improving local microcirculation through capillary dilation. Higher doses of salvianolic acid B not only prevent glucocorticoid-induced osteopenia but also increase cancellous bone mass and thickness, which is associated with an increase in marrow bone morphogenetic protein expression, inhibition of adipogenesis, and a further increase in microvessel diameters. Information on the dose-response relationship can provide important information for dose selection in clinical studies.

Accumulating evidence from cell and animal models has demonstrated that herbal medicines and phytochemicals have promising pro-angiogenic effects. However, more rigorously designed randomized controlled clinical trials should be conducted to verify their effectiveness and to clarify our understanding of their therapeutic potential. Relatively few randomized controlled trials have been conducted on herbal medicines for several reasons. First, the registration of certain herbal products in certain countries including China, Japan, Korea, and India does not require data from clinical trials if their formulations have been used for a long time. This is because they are considered safe and effective. Second, prescriptions containing herbal medicines are usually used for treatment. Clinical trials involving the use of a single herb are rare. Therefore, clinical data for specific herbs are usually unavailable. Third, quality control of herbal products is difficult, which makes the governmental authority or regulatory body unwilling to issue certificates for conducting clinical trials.

Future studies are required to design a dosing regimen, establish a delivery route, and demonstrate the safety of use. Regarding the phytochemicals mentioned in this review, ginsenosides, *Panax notoginseng* saponin, and astragaloside are safe and tolerated in humans (Xu et al., 2013; Xu et al., 2015; Fan et al., 2020). Clinical data regarding the safety of calycosin and ilexsaponin A1 are not available; however, in long-term clinical use, total glucosides of peony have produced no severe adverse reaction (Zhou et al., 2020). When patients use a combination of salvianolic acids and basic Western medicine therapy to treat acute cerebral infarction, salvianolic acids increase the occurrence of adverse events, such as headache, dizziness, hemorrhage of the digestive tract or skin, and mucosa, liver, or kidney injury. However, these adverse effects can be easily controlled or eliminated after the termination of salvianolic acid treatment (Xin et al., 2020). The use of tanshinones in pregnancy should be pursued with caution because they increase the drug permeability of the placental barrier (Zhou et al., 2019). Ferulic acid is a low-toxicity phytochemical, but it can aggravate renal cancer in rats with diabetes mellitus and renal cancer, and it causes renal damage after long-term treatment of chronic kidney disease (Li et al., 2021b). In addition, a meta-analysis of randomized controlled trials concluded that puerarin injection causes mild adverse reactions including nausea, vomiting, gastrointestinal discomfort, facial flushing, dizziness, and allergy (Zheng et al., 2017). In addition, some potential problems should be considered when pro-angiogenic therapy is applied. For instance, it is not known whether accelerated plaque angiogenesis leads to plaque rupture. Moreover, it remains unclear whether the use of pro-angiogenic agents increases the risk of angiogenesis-borne diseases, such as tumor formation and metastasis. Therefore, further studies are required.

## Conclusion

**FIGURE 3:**
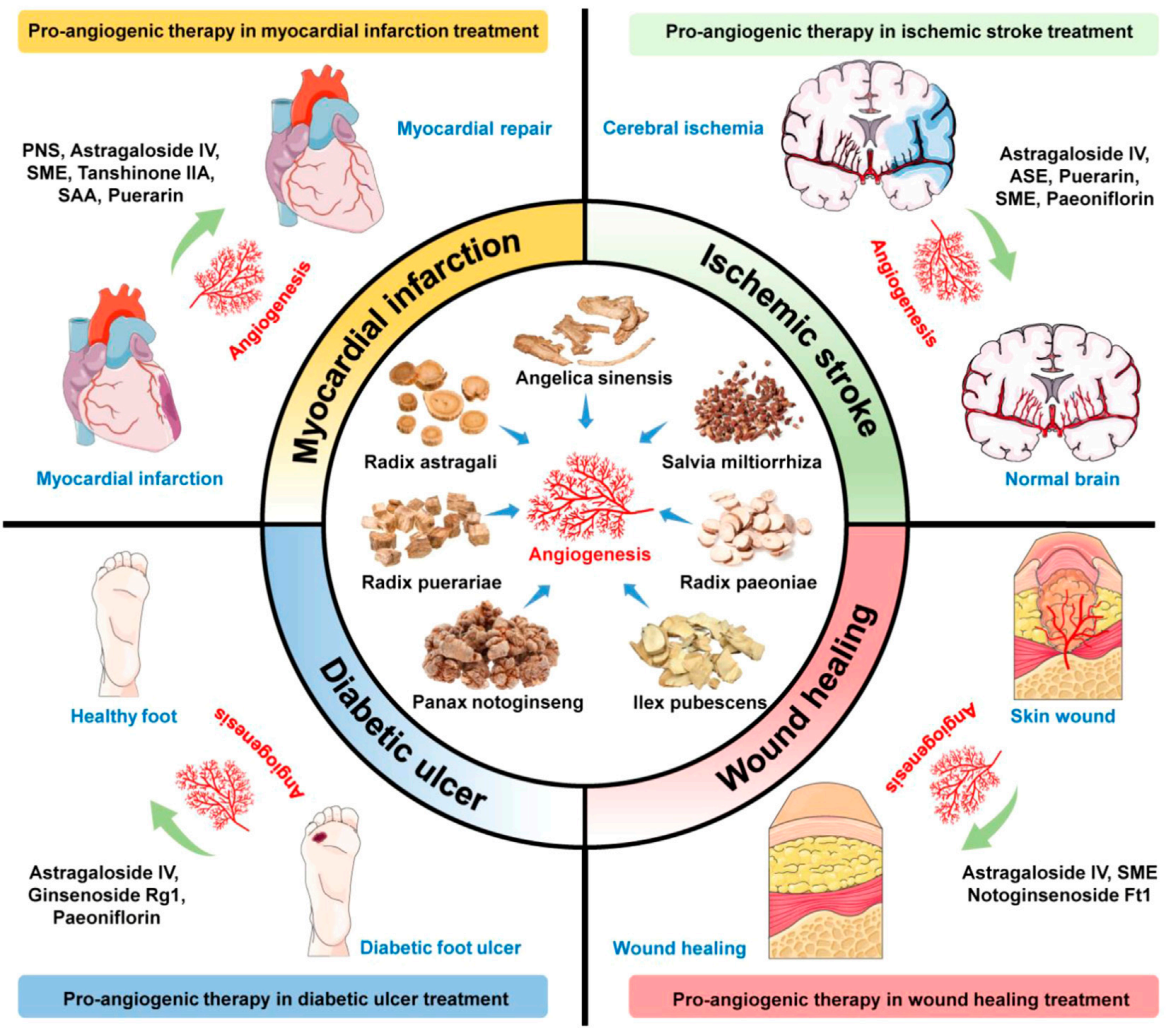
Summarized findings of pro-angiogenic phytochemicals for ischemic diseases, tissue repairing, and wound healing. Bioactive compounds or crude extracts of *Panax notoginseng*, Radix Astragali, *Salvia miltiorrhiza*, Radix Paeoniae, *Ilex pubescens*, *Angelica sinensis*, and Radix puerariae with pro-angiogenic activities show potential applications in treating ischemic stroke, myocardial infarction, diabetic ulcer, and wound healing. PNS, *Panax notoginseng* saponins; SME, *Salvia miltiorrhiza* extract; SAA, Salvianolic acid A; ASE, *Angelica sinensis* extract.

This study focuses on the potential use of herbal medicine and bioactive ingredients for the treatment of vascular insufficiency-related diseases such as ischemic cardiovascular and cerebrovascular disorders, and wound healing and tissue repair (Figure 3). The most frequently studied pro-angiogenic phytochemicals include ginsenosides from *Panax notoginseng*, astragalosides, calycosin from Radix Astragali, salvianolic acid B from *Salvia miltiorrhiza*, paeoniflorin from Radix Paeoniae, ilexsaponin A1 from *Ilex pubescens*, ferulic acid from *Angelica sinensis*, and puerarin from Radix puerariae. Their pro-angiogenic effects are promising and have been demonstrated in various cell and animal models. The major mechanisms of pro-angiogenic action are mostly related to VEGF-dependent signaling pathways. However, further pharmacokinetic and pharmacodynamic studies are warranted. In addition, there is still a need for more evidence-based confirmation through controlled and validated clinical trials. Traditional herbal medicine provides a fruitful resource for pro-angiogenic phytochemicals with different targets in the angiogenesis process. With the rapid development of various chromatographic separation technologies and different *in vitro*, *ex vivo*, and *in vivo* screening platforms, many pro-angiogenic compounds will be discovered. The application of medicinal chemistry and advancements in drug delivery technology has also accelerated the improvement of pharmacodynamic and pharmacokinetic properties of drug candidates. Therefore, the prospect of developing pro-angiogenic agents from herbal medicines and phytochemicals is promising.

## Glossary

Akt: protein kinase B
AMPK: 5′ adenosine monophosphate-activated protein kinase
Ang-1: angiopoietin-1
BAD: B-cell lymphoma-2 associated death
Bcl: B-cell lymphoma
BDNF: brain-derived neurotrophic factor
BM-MSCs: bone marrow-derived mesenchymal stem/stromal cells
BMSCs: bone marrow mesenchymal stem cells
CAM: chick embryo chorioallantoic membrane
Cdc42: cell division control protein 42 homolog
DAG: diacylglycerol
DO: distraction osteogenesis
EGF: epidermal growth factor
EGFR: epidermal growth factor receptors
eNOS: endothelial nitric oxide
EPCs: human endothelial progenitor cells
ER: estrogen receptor
ERK: extracellular signal-regulated kinases
FAK: focal adhesion kinase
FGF: fibroblast growth factor
G-CSF: granulocyte colony-stimulating factor
GSK-3β: glycogen synthase kinase-3β
HDAC5: histone deacetylase 5
HIF: hypoxia-inducible factor
HIF-1α: hypoxia-inducible factor-1α
HUVECs: human umbilical vein endothelial cells
IP3: inositol 1,4,5-trisphosphate
IRF-1: interferon Regulatory Factor 1
JNK: c-Jun N-terminal kinase
LAD: left anterior descending
MCAO: middle cerebral artery occlusion
MAPK: mitogen-activated protein kinase
MECs: microvascular endothelial cells
MMP: matrix metalloproteinase
mTOR: mechanistic target of rapamycin
N/A: not applicable
Nck: non-catalytic region of the tyrosine kinase adapter protein
NO: nitric oxide
PBMNCs: peripheral blood mononuclear cells
PDGF: platelet-derived growth factor
PI3K: phosphoinositide 3-kinase
PIP2: phosphatidylinositol 4,5-bisphosphate
PKC: protein kinase C
PKD1: protein kinase D1
PLC: phospholipase C
PNS: *Panax notoginseng* saponins
PPAR*γ*: proliferator-activated receptor γ
Raf: proto-oncogene serine/threonine-protein kinase
Shb: SH2 domain containing adapter protein B
SUMOylation: small ubiquitin-related modifier-mediated modification
TGF: transforming growth factor
VEGF: vascular endothelial growth factor
VEGFRs: vascular endothelial growth factor receptors
